# Lymph Node Yield in Gastrointestinal Cancer Surgery With or Without Prior Neoadjuvant Therapy: Protocol for a Systematic Review and Meta-analysis

**DOI:** 10.2196/35243

**Published:** 2022-04-28

**Authors:** Ulrich Ronellenfitsch, Nika Mathis, Juliane Friedrichs, Jörg Kleeff

**Affiliations:** 1 Department of Visceral, Vascular and Endocrine Surgery University Hospital Halle (Saale) Martin-Luther-University Halle-Wittenberg Halle (Saale) Germany

**Keywords:** lymph node yield, lymph node harvest, neoadjuvant therapy, neoadjuvant chemotherapy, neoadjuvant radiotherapy, surgery, resection, gastrointestinal cancer, chemotherapy, cancer

## Abstract

**Background:**

Lymph node yield is the number of lymph nodes retrieved during oncological resection and histopathologically identified in the resection specimen. It is an important surrogate parameter for assessing the oncological radicality of the resection of gastrointestinal carcinomas, as well as a prognostic factor in these diseases. It remains unclear if and to what extent neoadjuvant chemotherapy, radiotherapy, or chemoradiotherapy, which have become established treatments for carcinoma of the esophagus, stomach, and rectum and are increasingly used in pancreatic carcinoma, affect the lymph node yield.

**Objective:**

This systematic review with meta-analysis is conducted with the aim of summarizing the available evidence regarding the lymph node yield, an oncological surrogate marker, in patients with gastrointestinal carcinomas undergoing surgery after neoadjuvant therapy compared to those undergoing surgery without neoadjuvant therapy.

**Methods:**

Randomized and nonrandomized studies comparing oncological resection of esophageal, stomach, pancreatic, and rectal carcinoma with and without prior neoadjuvant therapy are eligible for inclusion regardless of study design. Publications will be identified with a defined search strategy in 2 electronic databases: PubMed and Cochrane Library. The primary endpoint of the analysis is the number of lymph nodes identified in the resected specimen. Secondary endpoints include the number of harvested metastatic lymph nodes, operation time, postoperative complications, pathological TNM staging, and overall and recurrence-free survival time. Using suitable statistical methods, the endpoints between patients with and without neoadjuvant therapy, as well as in defined subgroups (neoadjuvant chemotherapy, radiotherapy, or chemoradiotherapy; and patients with esophageal, gastric, pancreatic, or rectal cancer), will be compared.

**Results:**

The literature search and data collection started in October 2021. Results are expected to be published in mid-2022.

**Conclusions:**

This meta-analysis will provide the most up-to-date and complete summary of the evidence on an association between neoadjuvant therapy and lymph node yield in gastrointestinal cancer surgery. The underlying hypothesis is that neoadjuvant therapy decreases the number and size of lymph nodes through lymphocyte depletion and radiation-induced fibrosis, thus leading to a lower possible lymph node yield. The findings of the meta-analysis will show if this hypothesis is supported by evidence.

**Trial Registration:**

PROSPERO CRD218459; https://www.crd.york.ac.uk/prospero/display_record.php?ID=CRD42021218459

**International Registered Report Identifier (IRRID):**

DERR1-10.2196/35243

## Introduction

### Background

The TNM system for the classification and staging of malignant tumors in its current, eighth edition allows for prognostic statements about malignant tumor diseases depending on, among other things, the extent of lymph node involvement [1]. The N category in the TNM classification of gastrointestinal carcinomas is defined by the number of regional lymph nodes with histologically confirmed tumor invasion. Lymphadenectomy—the systematic resection of the regional lymphatic tissue and lymph nodes—is used for both therapeutic and staging purposes. Lymph node yield is the number of lymph nodes retrieved during oncological resection and histopathologically identified in the resection specimen. To allow for a valid statement about the number of affected lymph nodes, it is crucial that the lymph node yield is high—that is, that all regional lymph nodes are removed and identified in the subsequent histopathological examination. Therefore, treatment guidelines often stipulate a minimum number of lymph nodes to be removed and histopathologically analyzed. For example, regarding the surgical treatment of colorectal cancer, the current German S3 guideline specifies that 12 or more lymph nodes be removed and examined [2]. This is supported by the European Society for Medical Oncology Clinical Practice Guidelines for diagnosis, treatment and follow-up of rectal cancer [3] and the American Society of Colon and Rectal Surgeons Clinical Practice Guidelines for the Treatment of Colon Cancer [4].

However, some studies suggest that neoadjuvant chemotherapy, radiotherapy, or chemoradiotherapy, which have become established treatments for carcinoma of the esophagus, stomach, and rectum, and are increasingly used in pancreatic carcinoma, lower the lymph node yield in the case of colorectal cancer [5-9]. The mechanisms of this lower lymph node yield after neoadjuvant therapy could be based on lymphocyte depletion and radiation-induced fibrosis of the stroma, which lead to a reduction in the size of the lymph nodes and thus complicate their surgical and histopathological identification. Moreover, the occurrence of stromal atrophy and adipocytic replacement during therapy makes lymph node identification more difficult and can also contribute to a lower lymph node yield [10,11]. This mechanism has been shown in particular for radiotherapy and less so for chemotherapy [6]. Lastly, although there is no higher-level evidence supporting this hypothesis, differences in the surgeon’s approach for lymph node dissection of patients with or without prior neoadjuvant therapy—either more or less aggressive—may be of importance [7].

Differences in the lymph node yield could possibly lead to understaging of the N category in the TNM classification. This can affect the expected prognosis of the disease and thus have consequences for the decision for or against adjuvant therapy.

### Objective

Based on these considerations, it has become clear that the lymph node yield should play a major role in decisions regarding the therapy of malignant tumor diseases. The existing evidence on the effect of neoadjuvant therapy on the lymph node yield will be summarized in this systematic review with meta-analysis. The primary aim is to compare the lymph node yield of resections in esophageal, gastric, pancreatic, and rectal carcinoma after neoadjuvant chemotherapy, radiotherapy, or chemoradiotherapy with the lymph node yield after upfront resection. The secondary aim is to compare the lymph node yield in defined subgroups of patients (neoadjuvant chemotherapy, radiotherapy, or chemoradiotherapy; and patients with carcinoma of the esophagus, stomach, pancreas, or rectum) and to assess secondary outcomes such as the number of metastatic lymph nodes, the lymph node ratio, the incidence of postoperative complications, and postoperative survival time.

This report contains the protocol of the review.

## Methods

This protocol is reported according to the recommendations of the PRISMA-P (Preferred Reporting Items for Systematic Review and Meta-Analysis Protocols) 2015 statement [12]. The pertinent checklist can be found in Multimedia Appendix 1.

### Eligibility Criteria

The eligibility criteria for studies to be included in the systematic review and meta-analysis are shown in Textbox 1.

**Eligibility criteria.**

- The study includes patients in whom a carcinoma of the esophagus, stomach, pancreas, or rectum was resected oncologically (ie, with systematic lymphadenectomy).
- The study includes at least one group of patients who underwent neoadjuvant therapy (chemotherapy, radiotherapy, or chemoradiotherapy) prior to surgery and one group of patients who underwent upfront surgery (surgery without prior neoadjuvant therapy).
- The study reports the lymph node yield (the number of resected lymph nodes) for study participants.
- There is no limitation regarding study design if the above criteria are met.
- The abstract and full text of the study are available in English, German, Russian, Italian, Spanish, or French.

### Information Sources and Search Strategy

The electronic literature databases PubMed and Cochrane Library will be searched through their respective online search engines using a defined search strategy (Multimedia Appendix 2). The search will be performed on studies published between the databases’ inception and the cutoff date (October 8, 2020). Moreover, the reference lists of included articles will be manually searched.

### Data Management

The abstracts of the publications identified by the search strategy will be uploaded to the web application Rayyan QCRI (Rayyan Systems Inc) [13] to perform the study selection. Data extracted from the single studies will be stored in a standardized spreadsheet and will subsequently be transferred into the review software RevMan (version 5.3; The Cochrane Collaboration) [14].

### Selection Process

The abstracts of the studies identified by the literature search will be read by two independent reviewers to determine whether the studies meet the eligibility criteria. If a final assessment is not possible based on the abstract alone, the assessment will be based on the full text of the publication. A study is included or excluded from the systematic review based on a unanimous decision from both reviewers. If no agreement can be reached between the two reviewers, a third independent reviewer will act as an arbiter in the selection process. Duplicates and multiple reports of the same study will be identified and either excluded or collated so that each study, rather than each report, will be the unit of interest in the review. The record selection process will be recorded in a PRISMA (Preferred Reporting Items for Systematic Reviews and Meta-Analyses) flow diagram.

### Data Collection Process

A standardized data collection form will be used for the collection of study characteristics and outcome data. The form will be piloted on at least one study included in the review. A review author will independently extract the study characteristics and results from the selected studies.

### Data Items

From the full texts of the selected publications, the data shown in Textbox 2 will be collected for the overall study population and the defined subgroups, if available.

**Data to be collected from selected publications.**

- General information on the publication: title, author(s), date of publication, status of publication, journal in which the manuscript was published, language of the publication, funding of the study
- Study design
- Disease for which the study participants were treated (carcinoma of the esophagus, stomach, pancreas, or rectum)
- Patient characteristics: sex, age, American Society of Anesthesiologists (ASA) physical status [15], Eastern Cooperative Oncology Group (ECOG) Performance Status [16]
- Pretherapeutic clinical TNM stage
- Description of the surgical approach(es)
- Possible neoadjuvant therapy:
  Chemotherapy
  Radiotherapy
  Chemoradiotherapy
- Lymph node yield during resection (the total number of histopathologically identified lymph nodes in the resection specimen)
- Positive lymph nodes (the number of lymph nodes in the resection specimen with histopathological confirmation of tumor invasion)
- Lymph node ratio (the number of positive lymph nodes divided by the lymph node yield)
- Duration of the operation
- Postoperative complications (if available, according to the Clavien-Dindo classification [17])
- Pathological TNM stage (from resection specimen)
- Overall survival time (using the maximum available follow-up from the single studies)
- Disease-free survival time (using the maximum available follow-up from the single studies)

### Outcomes and Prioritization

A meta-analysis will be conducted for the primary and secondary outcomes shown in Textbox 3.

**Primary and secondary outcomes for meta-analysis.**

**Primary outcome**

• Lymph node yield during resection

**Secondary outcomes**

• Positive lymph nodes

• Lymph node ratio

• Duration of the operation

• Postoperative complications (if available, according to the Clavien-Dindo classification [17])

• Pathological TNM stage

• Overall survival time

• Disease-free survival time

### Risk of Bias in Individual Studies

The risk of bias of the individual studies will be estimated according to their respective study design. For nonrandomized studies, the Risk of Bias in Nonrandomized Studies of Interventions (ROBINS-I; The Cochrane Collaboration) tool will be used. Prior to assessment, an emulated ideal randomized controlled trial aiming to answer the research question will be conceived. This trial will serve as a risk of bias reference against which the selected studies will be compared. For randomized trials, the Risk of Bias tool for randomized trials (RoB 2; The Cochrane Collaboration) will be used. A full description of these tools can be found in the Cochrane Handbook for Systematic Reviews of Interventions [18,19]. The domains of bias considered for each study design are shown in Textboxes 4-5.

Specifically for this meta-analysis, the following confounding domains will be addressed: pretherapeutic tumor stage, pretherapeutic physical status, and age. These domains are used to decide whether a study participant undergoes neoadjuvant therapy or not. A specific cointervention to be considered as a potential source of confounding bias is the surgical approach, which could be related to the intervention received and is, at the same time, prognostic for the outcome of interest.

For each domain, the tools foresee “signaling questions” with response options of “yes,” “probably yes,” “probably no,” “no,” and “no information.” Based on the responses, the risk of bias for each domain will be judged as “low,” “moderate,” “serious,” “critical,” or “no information” in ROBINS-I and “low risk of bias,” “some concerns,” or “high risk of bias” in RoB 2. The risk of bias for the single domains will then be used to ascertain an overall risk of bias for the study according to Table 1.

**Domains of bias considered for nonrandomized studies.**

**Preintervention domains**

• Bias due to confounding

• Bias in selection of participants into the study

**At-intervention domain**

• Bias in classification of interventions

**Postintervention domains**

• Bias due to deviations from intended interventions

• Bias due to missing data

• Bias in measurement of the outcome

• Bias in selection of the reported result

**Domains of bias considered for randomized trials.**

- Bias arising from the randomization process
- Bias due to deviations from intended interventions
- Bias due to missing outcome data
- Bias in measurement of the outcome
- Bias in selection of the reported result

**Table 1:** Risk of bias judgment according to the ROBINS-I and RoB 2 tool.

Overall risk of bias judgment	Interpretation	Criterion for nonrandomized studies according to the ROBINS-I^a^ tool	Criterion for randomized trials according to the RoB 2^b^ tool
Low risk of bias	The study is comparable to a well-performed randomized trial.	The study is judged to be at low risk of bias for all domains for this result.	The trial is judged to be at low risk of bias for all domains for this result.
Moderate risk of bias (ROBINS-I)/some concerns (RoB 2)	The study appears to provide sound evidence for a nonrandomized study but cannot be considered comparable to a well-performed randomized trial.	The study is judged to be at low or moderate risk of bias for all domains.	The trial is judged to raise some concerns in at least one domain for this result, but not to be at high risk of bias for any domain.
Serious risk of bias (ROBINS-I)/high risk of bias(RoB 2)	The study has one or more important problems.	The study is judged to be at serious risk of bias in at least one domain, but not at critical risk of bias in any domain.	The trial is judged to be at high risk of bias in at least one domain for this result. OR The trial is judged to have some concerns for multiple domains in a way that substantially lowers confidence in the result.
Critical risk of bias (only ROBINS-I)	The study is too problematic to provide any useful evidence and should not be included in any synthesis.	The study is judged to be at critical risk of bias in at least one domain.	N/A^c^
No information (only ROBINS-I)	No information on which to base a judgement about risk of bias.	There is no clear indication that the study is at serious or critical risk of bias and there is a lack of information in one or more key domains of bias (a judgement is required for this).	N/A

^a^ROBINS-I: Risk of Bias in Nonrandomized Studies of Interventions.

^b^RoB 2: Risk of Bias tool for randomized trials.

^c^N/A: not applicable.

### Data Synthesis

The primary outcome (lymph node yield) will be reported separately for the intervention group (neoadjuvant therapy) and control group (upfront surgery) as a weighted mean with standard deviation. The groups will be compared using the weighted mean difference (and relative difference of standard deviation), for which 95% CIs will be calculated. A forest plot will be drawn. The same analysis will be done for the defined subgroups: patients who underwent neoadjuvant chemotherapy, neoadjuvant radiotherapy, or neoadjuvant chemoradiotherapy and patients with esophageal, gastric, pancreatic, or rectal cancer.

The secondary outcomes number of positive lymph nodes, lymph node ratio, and duration of the operation will be assessed in the same way. The secondary outcome postoperative complications will be dichotomized (grade 1 and 2 vs grade 3a and higher, according to the Clavien-Dindo classification [17]). The incidence of severe complications (grade 3a and higher) per group will be determined and compared using the chi-square test and a forest plot. The rates for the secondary outcomes overall and disease-free survival at 1, 3, and 5 years will be compared using weighted rates and a forest plot. The histopathological tumor stage (pathological TNM) will be qualitatively described for the groups.

Sensitivity analyses will be conducted according to ascertained risk of bias as described above. For these, all studies with a high or serious risk of bias will be excluded and the analyses of the primary outcome, as described above, will be conducted.

The *I*^2^ statistic, the *P* value from the chi-square test, and the between-study heterogeneity (τ^2^) will be used to assess heterogeneity among the studies in each analysis. If substantial heterogeneity (greater than 50%) is identified, reasons for this will be sought by performing subgroup analyses considering the specified subgroups and the causes of heterogeneity. Heterogeneity will also be assessed by evaluating whether there is good overlap of the confidence intervals. Any statistical heterogeneity will be taken into account when interpreting the results.

To assess possible publication bias, if the number of included studies is sufficient, we will create a funnel plot using the primary outcome and evaluate funnel asymmetry with Begg and Egger tests for continuous data [20,21] or Peters test for binary data [22].

### Assessing the Strength of the Body of Evidence

A “summary of findings” table will be created using the 5 Grading of Recommendations, Assessment, Development and Evaluations considerations (study limitations, consistency of effect, imprecision, indirectness, and publication bias) to assess the quality of the body of evidence—based on the studies that contributed data to the meta-analyses for each outcome—classifying it as high, moderate, low, or very low. The methods and recommendations described in the Cochrane Handbook for Systematic Reviews of Interventions will be used [23].

### Ethical Considerations

The ethical committee of the Medical Faculty of the Martin-Luther University Halle-Wittenberg waived this study from the need for ethical approval because no data from individual patients will be used (reference number: 2021-003).

## Results

The literature search and data collection started in October 2021. Results are expected to be published in mid-2022.

## Discussion

### Aim and Hypothesis

This systematic review with meta-analysis is conducted with the aim of summarizing all available evidence regarding the lymph node yield, an oncological surrogate marker, in patients with esophageal, gastric, pancreatic, and rectal carcinoma undergoing surgery after neoadjuvant therapy compared to those undergoing surgery without prior neoadjuvant therapy. One hypothesis is that neoadjuvant therapy decreases the number and size of lymph nodes through lymphocyte depletion and radiation-induced fibrosis, thus leading to a lower possible lymph node yield. The findings of the meta-analysis will show if this hypothesis is supported by evidence.

### Comparison to Prior Work

This meta-analysis will provide the most up-to-date and complete summary of the evidence on an association between neoadjuvant therapy and lymph node yield in gastrointestinal cancer surgery. Numerous single studies have been published on the topic, but they have shown heterogeneous results. To date, a comprehensive analysis of all the available evidence has not been completed.

### Limitations

This review is limited by the available publications at the time of the search of the literature databases (PubMed and Cochrane Library). The search was performed on studies published between the databases’ inception and the cutoff date (October 8, 2020) and is therefore limited. There is the possibility of publication bias. Moreover, the literature search is expected to identify mostly nonrandomized single studies for inclusion into the meta-analysis, which might cause bias in the results. Treatment protocols regarding neoadjuvant therapy and surgery will most likely vary between the single studies, which might lead to heterogeneous results.

## Appendix

Multimedia Appendix 1 PRISMA-P (Preferred Reporting Items for Systematic Review and Meta-Analysis Protocols) checklist.

Multimedia Appendix 2 Search strategy.

## Abbreviations

PRISMA: Preferred Reporting Items for Systematic Reviews and Meta-Analyses
PRISMA-P: Preferred Reporting Items for Systematic Review and Meta-Analysis Protocols
RoB 2: Risk of Bias tool for randomized trials
ROBINS-I: Risk of Bias in Nonrandomized Studies of Interventions

## References

[1] Brierley JD, Gospodarowicz MK, Wittekind C (2017). TNM Classification of Malignant Tumours, 8th Edition.

[2] (2019). Kolorektales Karzinom. Leitlinienprogramm Onkologie.

[3] Glynne-Jones R, Wyrwicz L, Tiret E, Brown G, Rödel C, Cervantes A, Arnold D, ESMO Guidelines Committee (2017). Rectal cancer: ESMO clinical practice guidelines for diagnosis, treatment and follow-up. Ann Oncol.

[4] Vogel JD, Eskicioglu C, Weiser MR, Feingold DL, Steele SR (2017). The American Society of Colon and Rectal Surgeons clinical practice guidelines for the treatment of colon cancer. Dis Colon Rectum.

[5] Doll D, Gertler R, Maak M, Friederichs J, Becker K, Geinitz H, Kriner M, Nekarda H, Siewert JR, Rosenberg R (2009). Reduced lymph node yield in rectal carcinoma specimen after neoadjuvant radiochemotherapy has no prognostic relevance. World J Surg.

[6] Mechera R, Schuster T, Rosenberg R, Speich B (2017). Lymph node yield after rectal resection in patients treated with neoadjuvant radiation for rectal cancer: A systematic review and meta-analysis. Eur J Cancer.

[7] Miller ED, Robb BW, Cummings OW, Johnstone PAS (2012). The effects of preoperative chemoradiotherapy on lymph node sampling in rectal cancer. Dis Colon Rectum.

[8] Baxter NN, Morris AM, Rothenberger DA, Tepper JE (2005). Impact of preoperative radiation for rectal cancer on subsequent lymph node evaluation: a population-based analysis. Int J Radiat Oncol Biol Phys.

[9] Ha YH, Jeong SY, Lim SB, Choi HS, Hong YS, Chang HJ, Kim DY, Jung KH, Park JG (2010). Influence of preoperative chemoradiotherapy on the number of lymph nodes retrieved in rectal cancer. Ann Surg.

[10] Fajardo LF (1994). Effects of ionizing radiation on lymph nodes. Front Radiat Ther Oncol.

[11] Shvero J, Koren R, Marshak G, Sadov R, Hadar T, Yaniv E, Konichezsky M, Feinmesser R, Gal R (2001). Histological changes in the cervical lymph nodes after radiotherapy. Oncol Rep.

[12] Moher D, Shamseer L, Clarke M, Ghersi D, Liberati A, Petticrew M, Shekelle P, Stewart LA, PRISMA-P Group (2015). Preferred reporting items for systematic review and meta-analysis protocols (PRISMA-P) 2015 statement. Syst Rev.

[13] Ouzzani M, Hammady H, Fedorowicz Z, Elmagarmid A (2016). Rayyan-a web and mobile app for systematic reviews. Syst Rev.

[14] Review Manager (RevMan). Cochrane.

[15] Abouleish AE, Leib ML, Cohen NH (2015). ASA provides examples to each ASA physical status class. ASA Newsletter.

[16] Oken MM, Creech RH, Tormey DC, Horton J, Davis TE, McFadden ET, Carbone PP (1982). Toxicity and response criteria of the Eastern Cooperative Oncology Group. Am J Clin Oncol.

[17] Dindo D, Demartines N, Clavien PA (2004). Classification of surgical complications: a new proposal with evaluation in a cohort of 6336 patients and results of a survey. Ann Surg.

[18] Sterne JA, Hernán MA, McAleenan A, Reeves BC, Higgins JP, Higgins JP, Thomas J, Chandler J, Cumpston M, Li T, Page MJ, Welch VA (2019). Assessing risk of bias in a non-randomized study. Cochrane Handbook for Systematic Reviews of Interventions, Second Edition.

[19] Higgins JP, Savović J, Page MJ, Elbers RG, Sterne JA, Higgins JP, Thomas J, Chandler J, Cumpston M, Li T, Page MJ, Welch VA (2019). Assessing risk of bias in a randomized trial. Cochrane Handbook for Systematic Reviews of Interventions, Second Edition.

[20] Begg CB, Mazumdar M (1994). Operating characteristics of a rank correlation test for publication bias. Biometrics.

[21] Egger M, Smith GD, Schneider M, Minder C (1997). Bias in meta-analysis detected by a simple, graphical test. BMJ.

[22] Peters JL, Sutton AJ, Jones DR, Abrams KR, Rushton L (2006). Comparison of two methods to detect publication bias in meta-analysis. JAMA.

[23] Schünemann HJ, Higgins JP, Vist GE, Glasziou P, Akl EA, Skoetz N, Higgins JP, Thomas J, Chandler J, Cumpston M, Li T, Page MJ, Welch VA (2019). Completing ‘Summary of findings’ tables and grading the certainty of the evidence. Cochrane Handbook for Systematic Reviews of Interventions, Second Edition.

