# Supplementary material for: Lymph Node Yield in Gastrointestinal Cancer Surgery With or Without Prior Neoadjuvant Therapy: Protocol for a Systematic Review and Meta-analysis
Source: JMIR Res Protoc. 2022 Apr 28;11(4):e35243. doi: 10.2196/35243 (PMC9100374; doi:10.2196/35243)
Supplement: Multimedia Appendix 2 [file resprot_v11i4e35243_app2.docx]

P

| (Esophag*[tw] **OR**  Oesophag*[tw] **OR**  gastr*[tw] OR  stomach*[tw] **OR**  pancreas*[tw] **OR**  rect*[tw])  AND  (Neoplasm*[tw] **OR**  Cancer*[tw] **OR**  Tumor*[tw] OR carcinoma*[tw]) |  |
| --- | --- |

AND

I

| (Neoadjuvant[tw] OR preoperative[tw] OR perioperative[tw])  **AND**  (Chemoradiotherapy[tw] **OR**  CRT[tw] **OR**  Radiotherapy[tw] **OR**  Chemotherapy[tw] OR irradiation[tw]) |  |
| --- | --- |

AND

C

| (primar*[tw]  OR initial*[tw]) AND resect*[tw] OR surgery[tw] |  |
| --- | --- |

AND

O

| Lymph*[tw] AND (node*[tw] OR nodal*[tw]) |  |
| --- | --- |
